# Study protocol of a clinical randomized controlled trial on the efficacy of an innovative Digital thErapy to proMote wEighT loss in patients with obesity by incReasing their Adherence to treatment: the DEMETRA study

**DOI:** 10.3389/fdgth.2023.1159744

**Published:** 2023-11-30

**Authors:** Gianluca Castelnuovo, Paolo Capodaglio, Ramona De Amicis, Luisa Gilardini, Sara Paola Mambrini, Giada Pietrabissa, Luca Cavaggioni, Giuseppina Piazzolla, Carlotta Galeone, Giacomo Garavaglia, Simona Bertoli, the DEMETRA Study Group, Amalia Bruno

**Affiliations:** ^1^ Clinical Psychology Lab, IRCCS Istituto Auxologico Italiano, Milan, Italy; ^2^ Department of Psychology, Catholic University of Milan, Milan, Italy; ^3^ Orthopaedic Rehabilitation Unit and Research Lab in Biomechanics, Rehabilitation and Ergonomics, IRCCS Istituto Auxologico Italiano, Piancavallo, Italy; ^4^ Department of Surgical Sciences, Physical and Rehabilitation Medicine, University of Turin, Turin, Italy; ^5^ International Center for the Assessment of Nutritional Status and the Development of Dietary Intervention Strategies (ICANS-DIS), Department of Food, Environmental and Nutritional Sciences (DeFENS), University of Milan, Milan, Italy; ^6^ Obesity Unit and Laboratory of Nutrition and Obesity Research, Department of Endocrine and Metabolic Diseases, IRCCS Istituto Auxologico Italiano, Milan, Italy; ^7^ Division of Nutritional Rehabilitation, IRCCS Istituto Auxologico Italiano, Piancavallo, Italy; ^8^ Department of Biomedical Sciences for Health, Università degli Studi di Milano, Milan, Italy; ^9^ Interdisciplinary Department of Medicine, School of Medicine, University of Bari “Aldo Moro”, Bari, Italy; ^10^ Bicocca Applied Statistics Center (B-ASC), Università degli Studi di Milano-Bicocca, Milan, Italy; ^11^ Biostatistics & Outcome Research, Statinfo, Milan, Italy; ^12^ Advice Pharma Group S.r.l., Information Technology Unit, Milan, Italy

**Keywords:** obesity, digital therapy, weight loss, diet, physical activity, cognitive–behaviour therapy, randomized control trial

## Abstract

Despite the increasing importance of innovative medications and bariatric surgery for the treatment of obesity, lifestyle interventions (diet and physical activity) remain the first-line therapy for this disease. The use of digital devices in healthcare aims to respond to the patient's needs, in order to make obesity treatment more accessible, so our study aims to assess the safety and efficacy of a Digital Therapy for Obesity App (DTxO) for achieving weight loss and its maintenance in patients affected with obesity undergoing an experimental non-pharmacological treatment. Here we present the study protocol of a prospective, multicenter, pragmatic, randomized, double-arm, placebo-controlled, parallel, single-blind study on obese patients who will be treated with a new digital therapy to obtain an improvement in their disease condition through the application of different simultaneous strategies (a dietary regimen and personalized advice program, a tailored physical exercise program, a cognitive–behavioural assessment and program, alerts and reminders, dedicated section on prescribed drugs intake, and chat and online visits with clinical professionals). We believe that DTxO will offer a promising intervention channel and self-regulation tool holding the potentiality to decrease treatment burden and treat more patients thanks to the partial replacement of traditional medical consultation with digital or telephone management, improving self- engagement and reducing the high demands the “obesity pandemic” for both patients and national health services in terms of time, cost, and effort.

**Clinical trial registration**: clinicaltrials.gov, identifier, NCT05394779.

## 1. Introduction

Obesity is defined as a multifactorial disease characterized by an excess of body fat which is associated with a significantly higher risk of multiple chronic diseases, for example diabetes mellitus, cardiovascular disease (CVD), depressive disorder, and different types of neoplasia (1–3). Moreover, obesity is associated with significant impairments in patients’ quality of life and even a reduction of life expectancy ranging from 5 to 20 years (4, 5).

Its prevalence has been globally increasing in the last 50 years to the extent that it is nowadays defined as an “obesity pandemic”, with the Global Burden of Disease (GDB) recently reporting that almost one third of world population can be listed as overweight or obese (5). Also in Italy obesity prevalence is rising, increasing by almost 30% in adult population in the last 30 years (6). Despite the increasing employment of medications and bariatric surgery for the treatment of obesity (7), lifestyle interventions (hypocaloric diet, physical activity, and cognitive-behavioral therapy) remain the first-line treatment (8). However, long term weight loss maintenance can be difficult both for metabolic readjustment and for the arduousness in lifestyle changes compliance, and patients often suffer weight regain following initial weight loss (9–11). Moreover, although drastic lifestyle changes usually lead to clinically significant and durable weight loss, they often require multiple in-person sessions with high demands for patients and national health services in terms of time, cost, and effort (12). Therefore, behavioral weight loss still has the unsolved issue of how to reduce healthcare intensity not affecting the regular social support, reliability, and feedback essentials to improve and preserve the “patient empowerment”. This recent concept has been proposed for managing diabetes (13) and is based on the concept that the patient is more encouraged to follow self-chosen behavioral adjustments than changes prescribed by others. Self-education with extensive lifestyle modifications and systematic self-monitoring provides positive and long-term effects on metabolic profile, together with quality of life, knowledge, and healthy behavior improvements (14).

It is in this context that digital health tries to respond to the patient's needs and makes the management of this condition more accessible (15). Scientific research has investigated different options of weight loss programs (computer, TV programs, smartphone applications), in order to meet patient's necessities and make obesity treatment more available (15). Smartphones indeed can be a valid option becoming a self-education device holding the capacity to reduce healthcare's workload and treat more people through the partial replacement of traditional medical consultation with digital or telephone management (16, 17). This approach seems the most convenient and accessible, considering the widespread use of smartphones among the population and the fact that weight-loss apps (Apps) can help reinforce dietary plan compliance, physical activity, and weight monitoring with the expedient of progress awareness through goals' achievement (18, 19). Consistent with these findings, our study aims to assess the safety and performance of a Digital Therapy for Obesity App (DTxO) for weight loss and its maintenance in patients affected with obesity undergoing an experimental non-pharmacological treatment. The primary objective is to evaluate the weight loss in patients using DTXO compared to control patients (Placebo App patients DTXO after a period of 6 and 12 months of DTXO use. The secondary objectives are:

1. To assess the degree of dietary recommendation adherence (Mediterranean dietary pattern questionnaire) and of improvement of food and nutrition knowledge (Food Knowledge Moynihan Questionnaire) of the two groups during our study
2. To evaluate the reduction of physical inactivity (reduction) (IPAQ Questionnaire), lower limbs physical function improvements (SPPB test) randomized in the two randomized group.
3. To measure the reduction of stress (DASS-21 scale), the increase in goodwill to approach therapy (URICA-28 scale), the increase in the (various) self-esteem in coping with life's challenges (GSES-10), the measure of Binge Eating Behaviour (BES-16 scale), the measure of emotion regulation problem (DERS-36 scale), the increase of quality of life (PGWBI questionnaire), the measure of sleep habits (PSQI) in each randomized group
4. To identify the level of acceptability in the use of DTxO after 12 months

## 2. Material and methods

### 2.1. Study design

**Figure 1:**
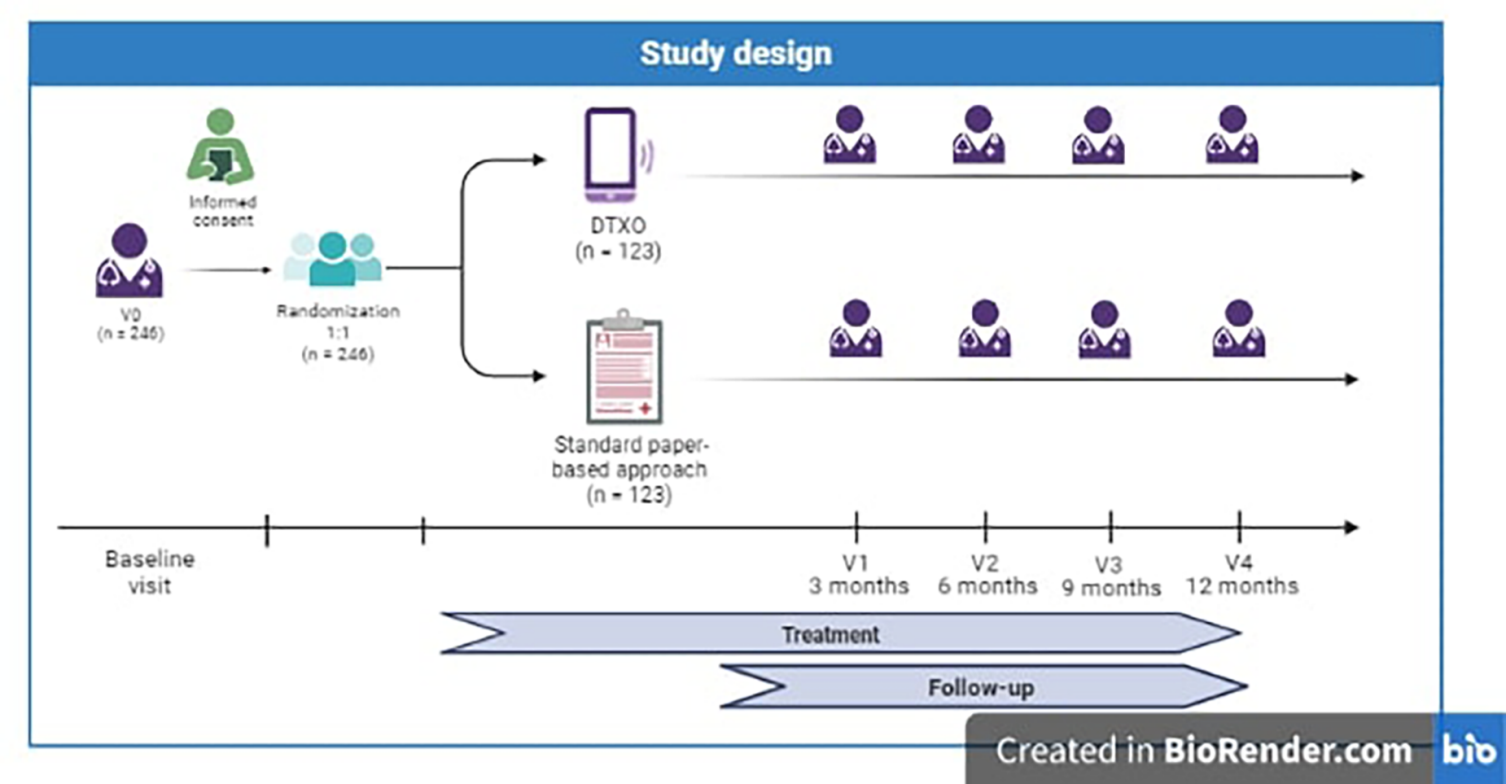
Study design of the DEMETRA study.

This is a prospective, multicenter, pragmatic, randomized, double-arm, placebo-controlled, parallel, single-blind study on obese patients treated with a new digital therapy (DTX Obesity) to obtain an improvement in their disease condition through the application of different simultaneous strategies (a dietary regimen and personalized advice program, a tailored physical exercise schedule, a cognitive-behavioural assessment and program, alerts and reminders, dedicated section on prescribed drugs intake, and chat and online visits with clinical professionals) (see Figure 1).

### 2.2. Study setting

The clinical centers involved in the trial are Istituto Auxologico Italiano and Azienda Ospedaliero-Universitaria Consorziale Policlinico Di Bari, specialized centers in obesity care. Both centers fully take part in trial activities, in the enrolling and take in charge of participants, implementation of interventions, and assessment of results. Heterogeneity is guaranteed both by the high number of patients, all enrolled in a comprehensive obesity outpatient clinic, and by the fact that the two centers are respectively in North and South of Italy, areas with different lifestyle and food habits.

### 2.3. Study duration

The estimated study duration is 16 months. Recruitment will last approximately 4 months. Each patient will be followed for approximately 12 months after the baseline visit.

### 2.4. Eligibility criteria

#### 2.4.1. Inclusion criteria

• Participants must be between 18 and 65 years of age at the time of informed consent.
• Informed consent form (Capability of giving informed consent, including compliance to requirements and restrictions listed in).
• BMI between 30.0 kg/m2 and 45 kg/m2.
• Participants must own, be prone to use technology of mobile Apps (tech-savvy) and be able to download the App described in the protocol.
• Italian language native speakers or foreign patients who fully understand Italian language, as the information and instructions for health programs will be in Italian.

#### 2.4.2. Exclusion criteria

• Diseases: recent cardiovascular event (<6 months), heart failure (class >II), GFR <60 ml/min, type 1 diabetes, cancer within the first 5 years; drug or alcohol abuse, active eating disorder or history of bulimia and anorexia nervosa, Binge Eating Scale score >27, psychiatric disorders not compensated, score ≥2 in the “Depression,” “Anxiety” and “Psychoticism” subscales of the Symptom Checklist-90-R; visual or vision impairments; secondary obesity, Edmonton Obesity Staging System score >4; bariatric surgery in the previous 2 years; referred pain to lower limb joints on the Numeric Rating Scale ≥5; recent (<6 months) weight loss ≥10%.
• Prior/Concomitant Therapies and other exclusions: Changed in pharmacological therapy with drugs that may affect eating behavior and/or energy metabolism during the last 3–6 months; participation in other weight-loss programs or in other clinical trials; known child-bearing women; any condition could the results of the study. Potentially interfering with the results of the study according to the investigator.

The presence of inclusion criteria and absence of exclusion criteria is investigated during the visit.

### 2.5. Recruitment

This study will include consecutive obese adult patients who self-referred to obesity centers seeking weight-loss treatment at the centers involved in the trial: patient enrolment will occur during a visit with an endocrinologist and a dietitian. Before any screening procedure, all patients must sign a written informed consent for the study.

### 2.6. Training

The training and experience necessary to use the device subject to pre-market clinical investigation (a device with pending CE marking and used in the investigation according to its intended use) were evaluated during the risk assessment of the device. It has been shown that the provision of information for safe use (User Manual) is sufficient for the healthcare professional user. Additionally, specific onboarding procedures for the patient have been determined as per the onboarding experience. Users will also be instructed to report any software malfunctions to the assistance service by emailing a dedicated assistance portal.

### 2.7. Randomization

Patients will be randomly assigned 1:1 to the intervention or comparison group according to a pre- defined, centralized randomization list. Randomization is indeed globally guaranteed between the two centers. To maintain an overall balancing between groups, we will perform block randomization (using random block sizes of 8 patients) (“plan”).

### 2.8. Blinding and unblinding procedures

Patients in the control arm will be provided with a placebo App to maintain patients' blindness to treatment allocation. Breaking the blind during the study (for a single subject) should be considered only when knowledge of the treatment assignment is deemed essential by the investigator due to immediate safety concerns or is considered essential for the immediate management of the subject, and should be discussed with the sponsor beforehand, if possible. It is the responsibility of the investigator to promptly document and explain any unblinding to the sponsor. Any unblinding will result in the discontinuation of subject participation from the study.

### 2.9. Study intervention

DTxO is an investigational therapeutic intervention for obese patients, under clinical validation with a randomized and placebo-controlled clinical trial for confirmatory purposes. DTxO is downloadable software for mobile devices (smartphone, tablet), classified as Class IIa Medical Device (MD), available for patients as an application (App) that integrates different non-pharmacological approaches. It is intended to improve weight loss, weight-loss maintenance, and overall health in patients with obesity by increasing their self-engagement, self-monitoring and adherence to dietary/exercise and behavioral programs. The App integrates different non-pharmacological approaches, engaging the patient through monitoring of her/his/them non-vital parameters, monitoring of patient diet and exercise, monitoring of patient psychological status, prescription of exercise and diet in a weight-loss program, and configurable data presentation charts for provision of additional information to professional users.

The Investigational Medical Device (IMD) consists of a Medical Web Application, also called Medical Dashboard, integrated into the electronic Case Report Form (eCRF), where the physician will collect patient's data necessary for enrolment and profile creation; and a Patient App, which will allow interacting with the patient to guide their behaviors, record therapeutic outcomes and improve therapeutic adherence.

The DTxO App will provide a dietary regimen with a personalized advice program, a tailored physical exercise program, a cognitive–behavioural assessment and support program, alerts and reminders, dedicated section on prescribed drugs intake and dietary and exercise program, chat and online visits with clinical professionals, and trophies to improve patient engagement. All these tools aim not only to weight loss, but they also help patients to improve their health and ameliorate their lifestyle.

The user downloads the App, creates their own account and starts to use it at baseline visit, by answering to psychological questionnaires and inserting food preferences. Thereafter, the app is synchronized with physician platform, so that a dietary regimen and a physical activity program is developed. The user then, helped by alerts and reminders, should daily use the app for consulting and filling in dietary, physical activity, and cognitive-behavioural sections, follow thee app's advice, be encouraged and self-monitor the situation. The App sends feedback and changes its advices, customizing them on the patient and going hand in hand with the constant use by the patient. The experimental arm will be compared with a control arm in which dietary and exercise programs will be delivered through a standard paper-based approach. The control arm will also be equipped with a placebo App, not customized on the habits and feedback of the patient, with no alerts and reminders nor chat or online visits.

### 2.10. Measurements and procedures

#### 2.10.1. Baseline visit (V0)

During the first visit, the patient will sign the informed consent form and will be evaluated for inclusion/exclusion criteria. Once the enrolment is confirmed, the patient is randomized 1:1 to either the experimental arm (DTx Obesity App) or control arm. The physician will explain to the patient how to download, access, and use the assigned App.

#### 2.10.2. Follow-up visit

After the baseline visit, the patient will follow the dietary, physical and psycho- behavioural programs suggested by the physician and will use the DTXO/placebo App as explained by the physician. Face-to-face follow-up visits are foreseen at 3 months (V1), 6 months (V2), 9 months (V3) and 12 months (V4) after enrolment. During each visit, the following assessments are performed:

• Physical examination
• Assessment of vital signs
• Laboratory blood tests
• Short Physical Performance Battery
• Assessment of patient's responses to psycho-behavioural questionnaires by a psychologist. If any psycho-pathological problem is detected, the patient is referred to a specialist and exits the study.
• Recording of concomitant medications
• Safety assessment (adverse events investigation)

#### 2.10.3. Telemedicine/remote contact

The Telemedicine visit and the remote contact between the patient and the physician will occur in specific situations related to weight- loss changes. This remote contact will allow the physician to understand the entity of the patient's symptoms and to manage them promptly. These utilities are part of the digital therapy: together with DTXO App, they will help patients to achieve treatment goals and assist them. To guarantee a proper assistance to all patients, control group was provided with email address and telephone number of enrolment outpatient clinic in case of need.

### 2.11. Study control

The efficacy of the DTXO App will be assessed with the comparison of results with a group of patients treated with a standard of care approach (paper-based, in-person) and a placebo App.

The placebo App will include only the non-medical modules of the digital therapy, for example, patient onboarding data and access, while it will not include any medical module; for example, the digital placebo App will not allow the input of the patient's reported clinical data nor provide indications or reminders about the therapy or diagnosis.

The placebo App has been introduced to make the experience of subjects in the control arm more similar to that of subjects in the experimental arm; moreover, data collection through the placebo app (questionnaires) will streamline the data collection process, avoiding manual data entry and reducing the possibility of entry errors. Through the placebo App, the patient will be asked to complete weekly the Diet Adherence questionnaire (NRS scale). No trend graph will be provided to the patient to monitor their performance, nor any personalized content will be displayed. standard clinical practice. The placebo App will be used only as a data entry tool for questionnaires.

Patients in the control group will be provided with a dietary plan and nutritional education and behavioral contents in paper format.

### 2.12. Statistical analysis

#### 2.12.1. Calculation of sample numerosity

Considering the primary endpoint, an 80% power and a two-sided type I error of 0.05 would be reached with a global sample size of 172 patients (86 per group) in order to be able to measure a 1.5 kg weight change after 6 months between the two groups, assuming an SD of 3.5 kg and a drop-out rate of 30%. We hence established a target of 246 patients considering a drop-out rate of 30%.

#### 2.12.2. Data analysis

We will perform descriptive analyses (at each visit) by computing categorical variables (percentages and frequencies) and continuous variables (mean and median values, standard deviations, quartiles, and extreme values). If useful, descriptive data will also be examined graphically through histograms and box-and-whisker plots. Appropriate statistical methods for comparisons between- group (i.e., interventional vs. control group) and within-group will be applied according to the nature of the variables (continuous or categorical variables), as specified in the following sections. % CI will not be finalized before the. Data analyses will be conducted using SAS version 9.4 (SAS Institute, Cary, NC, USA) statistical software.

## 3. Results

The advantage of the study is the possibility to provide simultaneous treatment strategies to improve the health status of obese patients. We expect that the concurrent application of these strategies and the possibility of customizing patients' activities based on their habits and feedback will allow greater benefits than the application of separate, no customized programs and a standard paper-based in- person approach. Both treatment arms will receive potential benefits by participating in this study.

The control arm will receive the current standard of care (paper-based approach and face-to-face follow-up visits) for obese patients with the addition of a placebo app. However, greater benefits are hypothesized in the experimental arm due to the possibility of customization of physical activity in accordance with the perceptions and feedbacks of the patient, the availability of notifications and reminders for drug intake (dosage, frequency) to improve treatment adherence, the availability of contents to support and favor compliance with the prescribed diet, the possibility to schedule an online visit or communicate with the clinical specialist via chat through the App, and the availability of multimedia psycho-behavioral content (motivational exercises, self-acceptance exercises, mindfulness exercises, interactive emotional eating exercises, self-efficacy exercise).

Differences between the DTxO and the App placebo are reported in Table 1.

**Table 1:** Differences between the dTxO and the App placebo.

	DTXO	App placebo
Diet		
Dietary plan and personalized suggestions’ alerts	X	
Dietary questionnaires		
Mediterranean-style eating questionnaire	X	X
Questionnaire food knowledge Moynihan questionnaire	X	X
Dietary program self- assessment		
Diet adherence (NRS scale)	X	X
Diet enjoyment (NRS scale)	X	
Hunger/satiety scale (NRS scale)	X	
Physical activity		
Physical activity plan	X	
Physical activity program self- assessment		
Level of physical activity questionnaire (IPAQ)	X	X
Fatigue perceived (NRS scale), Pain perceived (NRS scale)	X	X
Psycho-behavioural assignment		
Psychobehavioural self- assessment	X	X
DASS-21, URICA-28, GSES-10, BES-16, DERS-36, PGWBI, PSQI		
Recording of concomitant medications	X	
Telemedicine/remote contact	X	

## 4. Discussion

Considering the “obesity pandemic”, the benefits of lifestyle changing and the widespread use of smartphones among the population, the DTxO offers a promising intervention channel and self- regulation tool holding the potentiality to decrease treatment burden and treat more patients thanks to the partial replacement of traditional medical consultation with digital or telephone management. This approach seems the most convenient and accessible, since weight-loss apps (Apps) can help reinforce dietary plan compliance, physical activity, and weight monitoring with the expedient of progress awareness through goals' achievement (18, 19).

Different randomized controlled trials investigated the effects of digital health tools in weight-loss interventions in overweight and obese patients, showing the potential efficacy of self-monitoring of diet and physical activity with mobile phones combined with behavioural counselling or a newly developed, multifactorial, and daily-based personalized program for cognitive–behavioural therapy (CBT) conducted by a psychologist (20–22). One study also showed that the use of the digital health care service together with the support of a therapist online led to better results in anthropometric measures, for example body composition and body weight, and physiological indices and obesity-related psychological factors compared to the use of the app combined with self-care and no therapist intervention, thus suggesting that programs based on technological devices should be multidimensional and are undoubtedly most effective in combination with human support (23). Interestingly, literature data suggest that subjects using Apps related to health to record diet, physical activity, and health-related behaviours, gain significantly better results when the three dependent variables (i.e., lifestyle, physical activity, and eating behaviour) are combined compared with non-users (24).

Nevertheless, it was clear that app-based recording devices can be very appealing, considering the ascertained relationship between lifestyle and chronic diseases, since they can be very effective in preventative healthcare measures, increasing self-engagement, self- monitoring and adherence to dietary/exercise and behavioral programs. The App integrates different non-pharmacological approaches, engaging the patient through monitoring of her/his nonvital parameters, monitoring of patient diet and exercise, monitoring of patient psychological status, prescription of exercise and diet in a weight-loss program, and configurable data presentation charts for provision of additional information to professional users. Apps can be considered promising, since they can be harnessed for monitoring and motivation of patients to persevere in a healthy lifestyle (24).

## Members of the DEMETRA study group

Amalia Bruno, Antonina Orlando, Alessio Genovese, Federica Sileo, Marta Pellizzari, Marina Croci, Silvia Martinelli.
